# Supplementary material for: Extracellular Vesicle-Derived Protein File from Peripheral Blood Predicts Immune-Related Adverse Events in Gastric Cancer Patients Receiving Immunotherapy
Source: Cancers (Basel). 2022 Aug 28;14(17):4167. doi: 10.3390/cancers14174167 (PMC9454680; doi:10.3390/cancers14174167)
Supplement: Supplementary file 1 [file cancers-14-04167-s001.zip › cancers-1884503-SI.pdf]

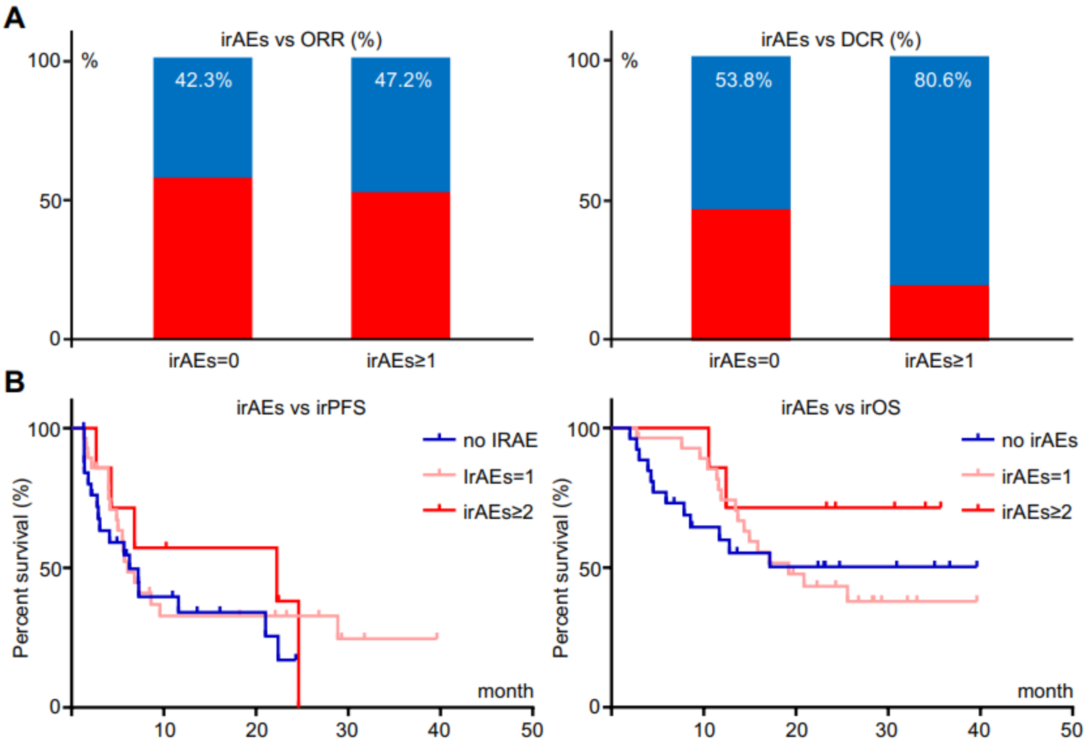

Figure S1. (**A**,**B**) The relationship between irAEs and ORR/DCR or irPFS of GC patients in discovery cohort.

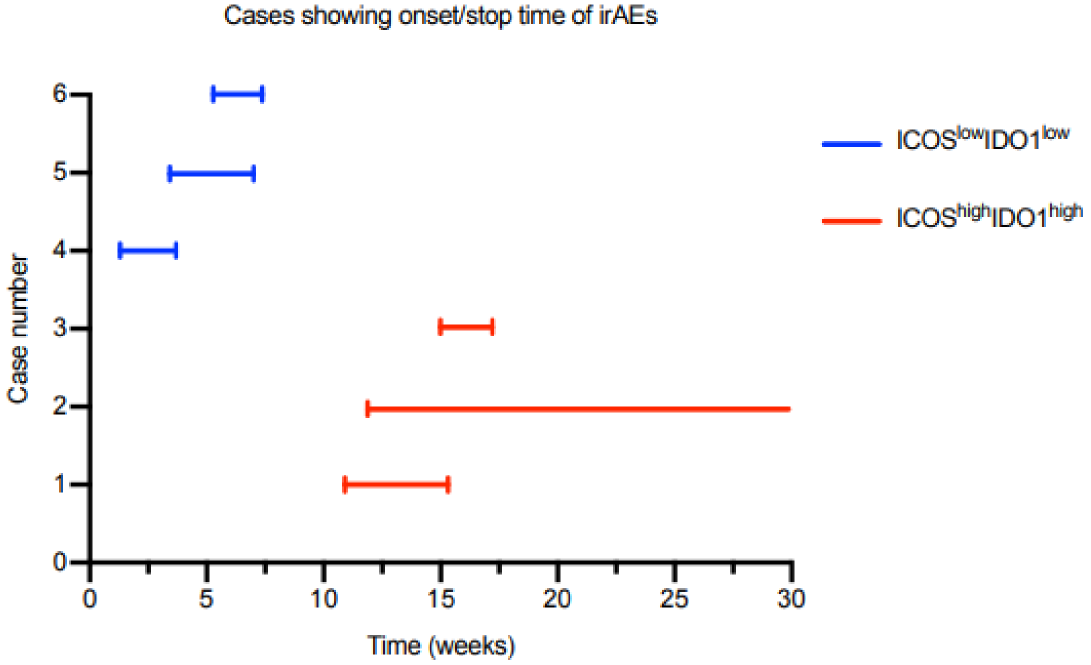

Figure S2. Dynamically showing onset/stop and duration of irAEs in ICOS/ IDO1 low and ICOS/ IDO1 high cases.

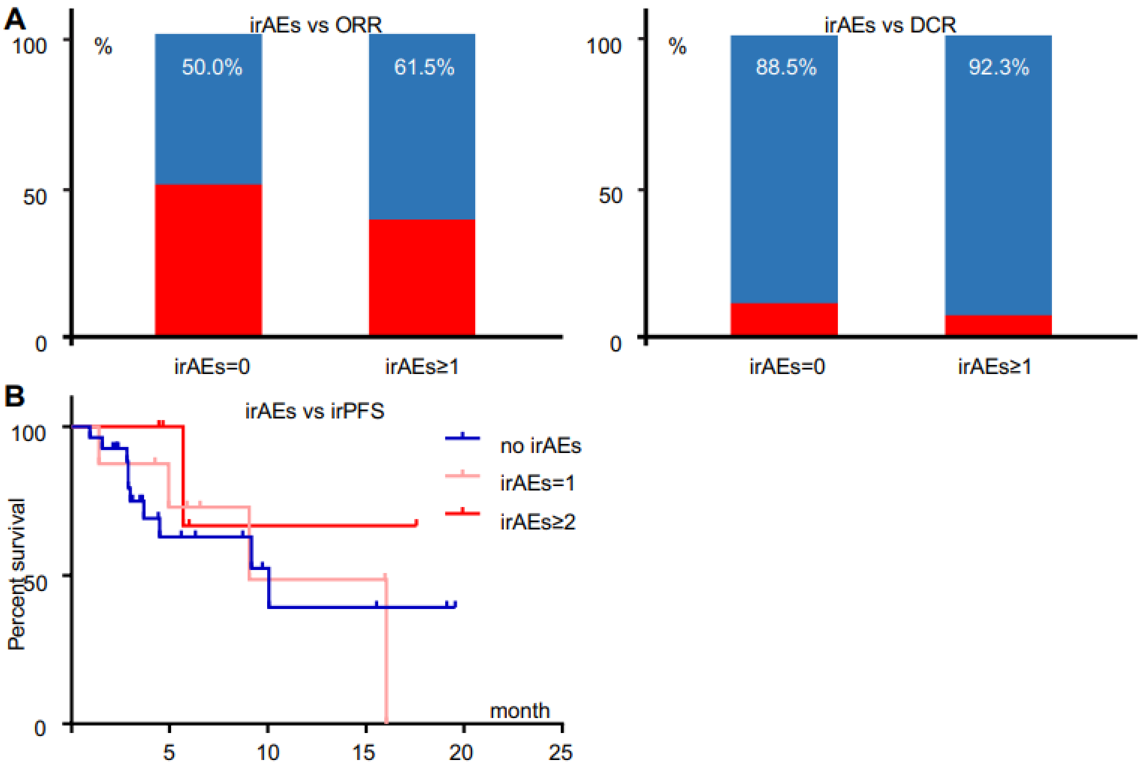

Figure S3. (**A**,**B**) The relationship between irAEs and ORR/DCR or irPFS of GC patients in validating cohort.
